# The potential risk of contralateral non-sentinel groin node metastasis in women with early primary vulvar cancer following unilateral sentinel node metastasis: a single center evaluation in University Hospital of Düsseldorf

**DOI:** 10.1186/s12905-020-01165-5

**Published:** 2021-01-12

**Authors:** Andreas Suhartoyo Winarno, Anne Mondal, Franca Christina Martignoni, Tanja Natascha Fehm, Monika Hampl

**Affiliations:** 1 Department of Obstetrics and Gynecology, University Hospital of Düsseldorf, Moorenstraße 5, 40225 Düsseldorf, Germany; 2 Department of Obstetrics and Gynecology, Franziskus Hospital Bielefeld, Kiskerstraße 26, 33615 Bielefeld, Nordrhein-Westfalen Germany; 3 Women’s Clinic on Schwanenmarkt, Düsseldorf, Germany

**Keywords:** Vulvar cancer, Sentinel lymph node biopsy, Non-sentinel lymph node, Inguinofemoral lymphadenectomy, Ipsilateral/contralateral lymph node metastasis

## Abstract

**Background:**

Since the introduction of sentinel node biopsy (SLNB) in unifocal vulvar cancer (diameter of < 4 cm) and unsuspicious groin lymph nodes, the morbidity rate of patients has significantly decreased globally. In contrast to SLNB, bilateral inguinofemoral lymphadenectomy (IFL) has been associated with increased risk of common morbidities. Current guidelines (NCCN, ESGO, RCOG, and German) recommend that in cases of unilaterally positive sentinel lymph node (SLN), bilateral IFL should be performed. However, two recent publications by Woelber et al. and Nica et al. contradict the current guideline, since a significant rate of positive non sentinel lymph nodes in IFL contralaterally was not observed [Woelber et al. 0% (*p* = 0/28) and Nica et al. 5.3% (*p* = 1/19)].

**Methods:**

A retrospective single-center analysis conducted in the University Hospital of Dusseldorf, evaluating vulvar cancer patients treated with SLNB from 2002 to 2018.

**Results:**

22.2% of women (n = 4/18) were found to have contralateral IFL groin metastasis after an initial diagnosis of unilateral SLN metastasis. The depth of tumor infiltrating cells correlated significantly and positively with the rate of incidence of groin metastasis (*p* = 0.0038).

**Conclusion:**

Current guideline for bilateral IFL should remain as the standard management. Therefore, this depth may be taken into account as an indication for bilateral IFL. The management of VC and SLNB should be performed in a high volume center with an experienced team in marking SLN and performing the adequate surgical procedure. Well conducted counseling of the patients outlining advantages but also potential oncological risks of this technique especially concerning rate of groin recurrence is critical.

## Background

Vulvar cancer (VC) is the fourth most common form of gynecological cancers. In 2016, 4.5 in 100,000 women/year, with a 5-year survival rate of 71% and mortality rate of 0.9 in 100,000 was reported in Germany, according to Robert Koch Institute’s data (RKI) [1]. A similar incidence is observed in the United States of America (USA) (surveillance, epidemiology, and end results program (SEER)) with an occurrence rate of 2.5 in 100,000 women/year, with a 5-year survival rate of 71% and mortality rate of 0.5 in 100,000 women/year [2].

In cases with locally confined histologically proven invasive (> 1 mm tumor depth) VC, complete resection of the tumor area ‘residual zero’ is the gold standard of current treatment [3–14]. Complete (radical) dissection of inguinofemoral LN or lymphadenectomy (IFL), was formerly the standard of groin staging. Following the publication of GRoningen INternational Study on Sentinel nodes in Vulvar cancer (GROINSS-V) in 2008 which showed that SLNB is safe in early vulvar cancer [6], the treatment modality was replaced in many countries with sentinel lymph node (SLN) biopsy (B) with radioactive tracer technetium 99^m^ nanocolloid (Tc-99^m^) and/or blue dye. The advantage of SLNB is the reduction in both morbidity (lymph cyst, lymphedema of leg, cellulitis or erysipelas) and mortality rates (septic shock due to wound infection and thromboembolism), shorter hospital stay and cost benefit implications [3–17]. However, SLN has limitations including unifocallity of the tumor, tumor size less than 4 cm, clinically unsuspicious LN in the groin and the need to inject radiocolloids before operating. Complex logistic and the expense of radioisotopes are also problematic. Moreover, blue dye as an alternative also has limitations such as inability to penetrate skin and fatty tissue and blue staining of the operation field [15, 16].

GROINSS-V also reported that the recurrence rate of SLNB was low with 2.3% (95% CI 0.6–5%) after a median follow up of 35 months in unifocal VC, with excellent 3-year survival rate 97% (95% CI 91–99%) and minimal morbidity SLNB compared with IFL. Wound break down was 11.7% versus 34% in SLNB; cellulitis 4.5% versus 21.3%; erysipelas 0.4% versus 16.2%; and lymphedema of the legs 1.9% versus 25.2% [6]. In the GOG-173 study of Levenback et al. [17], the false negative rate of SLNB was about 8.3%. The latest European expert panel recommends that SLN detection could be improved by use of indocyanine green (ICG) and additional application of SPECT/CT imaging can reduce the false negative results [18]. It is also worth noting that the recurrence rate of metastasis was 2.7% with SLNB and 1.4% with IFL [8, 17]. The survival rate, in the case of isolated groin lesion recurrence, was reported worser in women with primary local VC > 4 cm than < 4 cm in initial negative SLNs with 9% versus 5% respectively [8]. AGO-Care-1 cohort study showed that ˃ 60% of women with VC bigger than 4 cm had at least 1 metastatic LN. Therefore, the current recommendation is to use SLNB only for tumors less than 4 cm of diameter [19]. Many studies show that the amount of groin LN metastasis is a significant negative predictor for survival [20, 21]. Recurrences of metastatic LN in the groin are associated with high mortality rates [22].

Moreover, the procedure itself should be performed only in dedicated hospitals, by surgeons with specialty expertise and adequate number of procedures-per year [23].

In the case of SLNB with positive unilateral metastasis, current German guidelines recommend bilateral IFL as standard treatment [4]. European Society of Gynaecological Oncology (ESGO), Royal College of Obstetricians and Gynecologists (RCOG), and National Comprehensive Cancer Network (NCCN) are in accordance with the German guideline [2–4, 24, 25].

This retrospective single center study aims to evaluate whether the current guideline should remain as standard care or whether there is evidence to recommend the omission of a complete IFL resection of the contralateral groin in the case of positive unilateral SLN only.

## Methods

### Patients

**Fig. 1:**
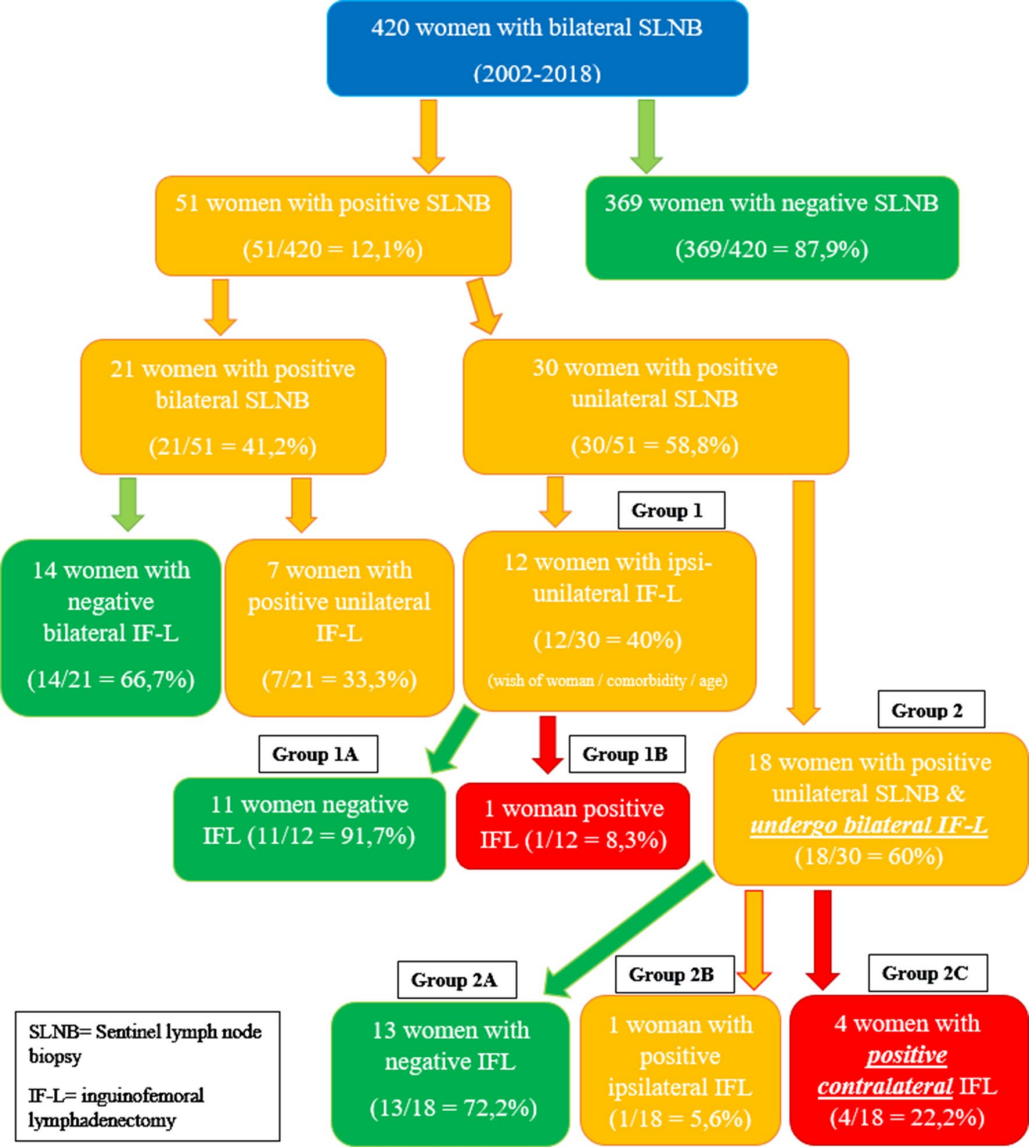
Patient selection for retrospective single-center data analysis

All patients were diagnosed with primary vulvar squamous cell carcinoma at the Obstetrics (O) and Gynecology (G) clinic in the University Hospital of Düsseldorf (UHD) between 2002 and 2018 (Fig. 1). The Ethics committee of the medical board of Heinrich Heine University approved the retrospective investigation of patients’ medical records (Reference Number 2019-491). Out of the 420 women who were evaluated, 369 women with negative metastasis of SLNB were ruled out. Of the remaining 51 women, 30 had unilateral SLNB metastasis and 21 had bilateral SLNB metastasis. Inclusion criteria for SLNB procedures were Stage IB, II VC and tumor size less than 6 cm without suspicious groin LN clinically. Exclusion criteria were multifocality, tumor size above 4 cm with highly suspicious groin LN, and distant metastasis at initial diagnosis or consent refusal of the patients due to the potential increased risks of groin recurrence. In the final analysis, we focused on women with positive SLN unilaterally.

### Removal of primary tumor and identification of sentinel node

Vulvar tumor was resected locally with ˃ 3 mm tumor free margin or partial/total vulvectomy. The surgical procedure was performed by one experienced vulvar surgeon with rare exception. If in the final histology report, tumor free margin was reported to be less than 3 mm, an additional resection was performed separately. Regional flaps for wound closure were used when indicated [4]. In pregnancy, primary tumor was resected immediately in any gestational age after punch biopsy confirmed malignancy.

One day before surgery, all patients underwent peritumoral intradermal injection of Tc-99^m^ at three, six, nine and twelve o’clock using a 27-gauge needle. An hour following the injection, a planar lymphoscintigraphy was performed with anterior and lateral static view. This procedure followed the GROINSS-V protocol adapted by German guideline [4, 6, 26–29]. However, a short protocol with dose reduction was chosen for pregnant women. SLNB procedure was performed after 14 weeks of pregnancy (WP). The administration of Tc-99m was lower than 100 MBq. An abdominal shield was used to protect the fetus from radiation in performing planar lymphoscintigraphy. These procedures were done 2 h before the SLNB operation [26–29].

On the day of surgery, a handheld gamma probe (Neoprobe GDS, BT Devicor Mammotomo, Cincinnati, OH, USA) was used to identify marked groin nodes bilaterally. In the case of SLN metastasis in final histology, IFL was further performed separately with patient consent. Pelvic node dissection was indicated in accordance with German guideline: more than two metastatic nodes or one metastatic node ˃ 5 mm or extracapsular spread.

### Histopathology

Pathological examination was performed in the Department of Histopathology at University Hospital of Düsseldorf. A standard protocol has been established for all sentinel node procedures which included frozen sections of LNs (not in all cases performed), hematoxylin and eosin staining, subsequent ultra-staging and immunohistochemistry with three sections per 5 mm, similar to the GROINSS-V study protocol [4, 6, 26–29].

### Classification

VC was classified into tumor (T), nodal (N), metastasis (M), grading (G), perineural (Pn), lymphovascular space (L) or blood vessel (V) infiltration and resection status (R) histologically. The International Federation of Gynecology and Obstetrics (FIGO) system was used for clinical staging.

### Statistics

**Fig. 2:**
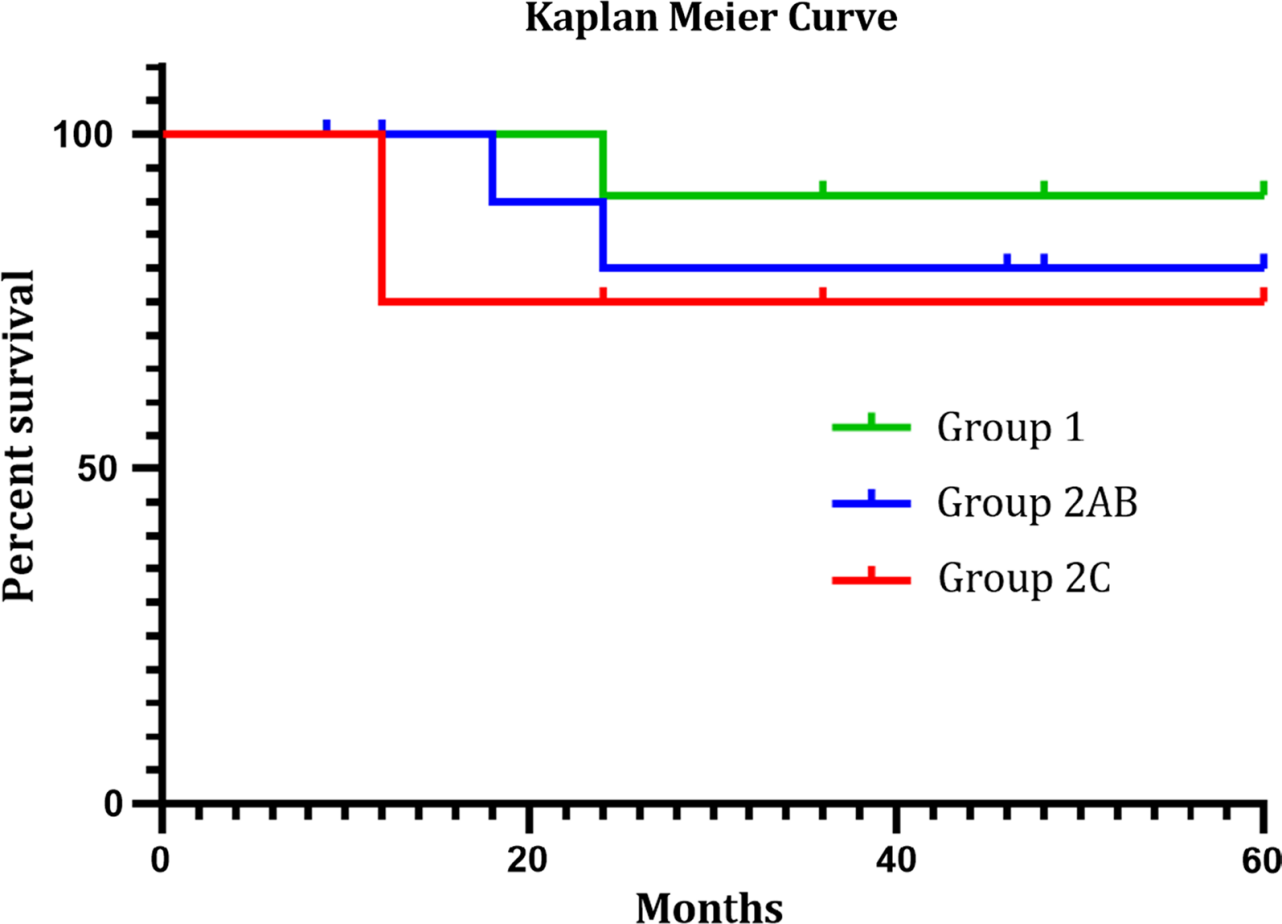
Kaplan Meier curve of patient’s survival rates. OS analysis results show 90.9% for group 1, 80% for group 2AB and 75% for group 2C. Group divisions can be seen in Fig. 1

All groups were analyzed using one-way ANOVA to determine their statistical significance. *p* values of < 0.05 was considered to be statistically significant. One-way ANOVA analysis was done with Microsoft Excel professional plus 2016 (Table 1). Graph prism 8.3 was used to analyze the overall survival (OS) using Kaplan Meier curve (Fig. 2). The minimum follow-up period of the patients was 12 months after initial diagnosis, also their initial diagnosis of VC was at about 6–8 years ago. Due to the retrospective nature of the study, 8 of 18 women were lost in follow up, due to changes in their home address, gynecologist and/or phone number. Attempts to contact all these cases were without success. In addition, some patients were lost in subsequent follow-up because they were examined by their local gynecologists or at the nearest hospitals.

**Table 1:** Patient, disease and treatment characteristics

SLNB	Group 1 (n = 12)	Group 2A&B (n = 14)	Group 2C (n = 4)	*p* value
Age (years)				0.793
Median (range)	50 (28–79)	52.5 (27–82)	55 (51–67)	
Primary vulvar tumor location				0.553
Midline	9 (75%)	11 (78.6%)	4 (100%)	
Lateralized	3 (25%)	3 (21.4%)	0	
Diameter (mm)				0.7645
Median (range)	15.5 (6.0–54.0)	19.5 (9.0–60.0)	22.5 (16.0–35.0)	
Diameter categories				0.246
< 20 mm	7 (58.3%)	7 (50%)	1 (25%)	
≥ 20 mm but < 40 mm	3 (25%)	5 (35.7%)	3 (75%)	
≥ 40 mm	2 (16.7%)	2 (14.3%)	0	
Depth (mm)				0.0038*
Median (range)	3.0 (1.8–6.0)	6.0 (2.0–15.0)	8.5 (5.0–23.0)	
Grade				0.410
1	–	–	–	
2	10 (83.3%)	11 (78.6%)	2 (50%)	
3	2 (16.7%)	3 (21.4%)	2 (50%)	
Radiotherapy	4 (33.3%)	6 (42.9%)	3 (75%)	
Chemotherapy	0	2 (14.3)	2 (50%)	
Local recurrence	1 (8.3%)	1 (7.1%)	1 (25%)	
Groin recurrence	1 (8.3%)	0	0	
	(fat tissue)			
Distant metastases	1 (8.3%)	0	1 (25%)	

A *p* value of less than 0.05 is considered to be statistical significant

*At depth of infiltrating tumor

## Results

Our data were collected from 420 women with early primary VC and bilateral SLNB from 2002 to 2018. Fifty-one women (12.1%) had either unilateral SLN metastasis (n = 30; 58.8%) or bilateral SLN metastasis (n = 21; 41.2%). Those with unilateral SLN metastasis (n = 30) had a median age of 51.5 years old (SD ± 14.4; max 82; min 27) and were further divided into two groups (Fig. 1):

### Group 1

Twelve women (n = 12/30; 40%) had ipsilateral IFL only, in accordance with the patient´s desire to avoid the increased morbidity of bilateral groin surgery and/or old age. Only one woman was diagnosed having an additional positive metastatic LN in IFL (1/12 = 8.3% (group 1B) and 11 women (11/12 = 91.7%) (group 1A) had no further metastatic LNs in IFL.

- One woman (n = 1/11; 9.1%) in group 1A suffered from local recurrence 6 months after initial diagnosis and she survived at 60 months’ follow-up.
- One woman (n = 1/11; 9.1%) in group 1A experienced VC recurrence in the fat tissue of the groin 18 months after initial diagnosis of ipsilateral IFL without further metastatic LNs. She had an initial diagnosis with 5.4 cm tumor diameter of focal VC. She received re-surgery and radiotherapy and still survived at 60 months’ follow-up examination.
- One woman (n = 1/11; 9.1%) in group 1A had 4.7 cm tumor diameter at initial diagnosis and a 5 mm left-sided SLN metastasis with extracapsular spread and due to their own decision only IFL on the ipsilateral side including left sided pelvic LN dissection was performed revealing no further metastatic LNs in IFL or iliac nodes. Therefore, radiotherapy was suggested for vulvar region (R1 resection, G3 tumor) and bilateral groin. Unfortunately, she was diagnosed with metastases in both lungs, liver and bone 15 months later. Her bronchial biopsy result was negative for p16 expression in tumor cells, whereas the VC has been positive for p16 expression suggesting a HPV induced vulvar cancer. Histologically, both tumors were squamous cancer cells. Therefore, she was suspected to have primary lung cancer in addition to her vulvar cancer. She received palliative radio-chemotherapy.
- There was only one woman (n = 1/12; 3.3%) with subsequent metastatic LN in ipsi-unilateral IFL. She had received radiotherapy to her right groin. She survived 24 months after initial diagnosis and then was lost in subsequent follow-up.

### Group 2

Eighteen women (n = 18/30; 60%) who received complete bilateral IFL were further divided into three subgroups:

A: Thirteen women (n = 13/18; 72.2%) had negative IFL results in both groins.

- Interestingly, a 30-year old woman from subgroup 2A was diagnosed with VC in her second pregnancy. Her clinical complaints were persistent itchiness, pain and ulceration of vulva. A punch biopsy showed keratinized squamous cell cancer. Therefore, removal of the vulvar ulcerative lesion was done at 7th weeks of pregnancy (WP) and SLNB at 19th WP revealing a unilateral metastatic SLN. She received bilateral IFL at 20^th^ WP. She subsequently had an uneventful pregnancy and delivered her baby via caesarean section at term. Her most recent examination, 60 months after initial diagnosis, at our outpatient clinic showed no sign of recurrence.
- Another 36-year old woman had similar complaints at 23rd WP and her punch biopsy result showed low-grade chronic inflammation, reactive squamous cell hyperplasia and hyperkeratosis. Fourteen months later, while still breastfeeding, VC was diagnosed with a non-keratinized squamous cell type of carcinoma located at the right labia minora with extension close to the clitoris. Standard surgery revealed a left-sided SLN metastasis of 3 mm with extracapsular tumor cell spread. Consecutive bilateral IFL did not find any further metastatic lymph nodes. As a result, she received radiotherapy on her left groin and follow-up examination at 60 months after initial diagnosis showed no sign of recurrence.
- A woman had 5.7 cm focal tumor diameter of initial diagnosis VC. The follow up was uneventful.

B: One woman (n = 1/18; 5.8%) had further metastatic lymph nodes in the ipsilateral IFL.

C: Four women (n = 4/18; 22.2%) had contralateral groin metastatic LN in IFL following unilateral SLN metastasis initially (Table 2).

- One woman (n = 1/4; 25%) from Group 2C developed mons pubis malignant squamous cell tumor. The focal tumor was at anterior fourchett between clitoris and urethra. 18 months later, radio-chemotherapy after surgical in toto removal of this tumor occured.

**Table 2:** Patient characteristics and pathological findings of four women with contralateral SLN metastasis following bilateral IFL

	Initial diagnosis	Smoker	Pathological results and disease chronology
1	2005 54 years’ old TZ = 2 cm TI = 5 mm BMI = 19.1	Yes	SLNB showed left-sided metastasis 2 mm. IFL showed right-sided metastasis 2 mm. Subsequently, she received radiotherapy of both groins 2011: squamous cell laryngeal cancer (negative p16) 2014: squamous cell pulmonary cancer (negative p16) 2019: still alive with no signs of recurrence
2	2015 56 years’ old TZ = 3,5 cm TI = 9 mm BMI = 29.8	No	SLNB showed right-sided metastasis 9 mm with infiltration of blood vessel (V1) IFL showed left-sided metastasis 7 mm with extra capsular tumor cells Pelvic lymphadenectomy showed no metastasis. Subsequently, she received bilateral radiotherapy of her inguinal regions 2019: still alive with no signs of recurrence
3	6/2016 67 years’ old TZ = 2,5 cm TI = 8 mm BMI = 28.8	Yes	2014: kidney transplantation (tacrolimus and mycophenolic acid) 6/2016: SLNB showed right-sided metastasis 3 mm. Complete IFL showed left-sided metastasis 3 mm Tacrolimus was changed into Everolimus. The patient received bilateral radiotherapy of her inguinal regions 8/2017: passed away due to lung metastasis of vulvar cancer
4	2018 51 years’ old TZ = 1,6 cm TI = 2,3 mm BMI = 25.6	Yes	SLNB showed right-sided metastasis 3 mm. IFL showed left-sided metastasis 8 mm. Pelvic lymphadenectomy showed no metastasis. Subsequently, she received bilateral radiotherapy of her inguinal regions 2019: recurrent vulvar cancer after 18 months (metastasis in fat tissue at mons pubis paramedian on the left side with infiltration into venous blood vessel). Surgery followed by radio chemotherapy was performed until December 2019

*TZ* tumor size, *TI* tumor infiltration, *BMI* body mass index

All of the 30 women (n = 30/51; 58.8%) with positive unilateral SLNB had pT1b VC, except for one with pT2. A majority of the women with positive unilateral SLNB (n = 24/30; 80%) suffered from anterior midline lesions between clitoris and urethra. The median size of tumors was 1.9 cm (SD ± 1.4 cm) and the median depth of tumor cell infiltration was 5 mm (SD ± 4.4 mm). Only one woman (n = 1/30; 3.3%) had posterior midline vulvar lesion. Five women (n = 5/30; 16.7%) had lateralized lesions. In contrast, all women with contralateral groin metastatic lymph nodes in IFL (subgroup 2C) had anterior midline lesions.

In this study, we would like to highlight the side effects and complications post-surgery from Groups 1 and 2. Thirteen women (n = 13/30; 43.3%) suffered edema of the foot and required lymphatic drainage therapy. Nine women (n = 9/30; 30%) developed lymph cysts and five women (n = 5/30; 16.7%) had erysipelas and required antibiotic therapy. The comparison from each group regarding the complications postoperatively is shown in Table 3. Three women (n = 3/30; 10%), one woman from each Group 1, 2A and 2C, developed local recurrence of VC.

**Table 3:** the postoperative complication from each group

	Ipsi-unilateral IFL Group 1	Bilateral IFL Group 2 A&B	Bilateral IFL Group 2C
Edema of legs	25% (n = 3/12)	42.8% (n = 6/14)	75% (n = 3/4)
Lymph cyst	25% (n = 3/12)	28.6% (n = 4/14)	50% (n = 2/4)
Erysipelas	25% (n = 3/12)	14.3% (n = 2/14)	0% (n = 0/4)

OS analysis was performed for all 30 women with unilateral SLN metastasis. The 5 years’ survival rates were 90.9% in Group 1, 80% in Group 2A/B and 75% for the four women in Group 2C.

## Discussion

Since the introduction of GROINSS-V study in 2008, SLNB of the groin has played a central role in the management of VC. Firstly, SLNB has reduced morbidity and mortality rates, whereas radical IFL has significant side effects [6]. Secondly, the necessity for IFL remains controversial in the case of positive unilateral SLNB, as to whether it should be done ipsilaterally or bilaterally [1–8]. This is due to the fact that when recurrent groin metastasis occurs, the survival rates of these patients decrease significantly [9–25, 30–33].The long-term follow-up of GROINSS-V showed that the 10-year disease-specific survival rates in the cases of local recurrence was reduced from 90.4 to 68.7% and in patients with positive SLNB from 77.7 to 44.6% [10].

A German study of Woelber et al. [7], showed in none of the cases of primary VC with positive unilateral SLN contralateral positive LN in consecutive bilateral IFL (0/28 cases, 0%). A Canadian study (Nica et al.) [8] reported that only 1 of 19 patients (5.3%) had a contralateral metastatic LN in IFL following unilateral SLN metastasis. But, two of their patients with positive unilateral SLNB had a groin recurrence (one located unilaterally and the other contralaterally) several months following negative IFL [8]. Therefore, they suggest it is reasonable to omit contralateral IFL in patients with unilateral SLN metastasis. Both studies are in contrast to our findings with 4/18 (22.2%) women with unilateral positive SLN diagnosed with contralateral positive LN in IFL. The reason for this discrepancy may be the fact, that in our study, the tumors of these four women were all located in the midline. Unfortunately, Woelber et al. and Nica et al. did not specify the location of the tumors, if they were midline or lateral [7, 8, 12].

Over the past decade, there has been an increasing trend for midline VC [34–37]. In our hospital, the overall percentage of VC located in the anterior fourchette area is approximately 60%. Four cases with contralateral IFL metastasis in our study had originated from midline lesions. Therefore, our data suggests if the patient has unilateral SLN metastasis, clinicians should offer radical bilateral IFL in case of midline tumors. This is the current recommendation in German guideline [4]. Our retrospective single-center study results with a rate of 22% of contralateral positive LN after unilateral positive SLN confirms that current guidelines are appropriate and should not be amended or changed, because our results suggest, that the risk of groin recurrences will be significant if the contralateral groin resection is omitted, also taking into account that none of the women with ipsilateral IFL only (12/30 women, own wish) developed a groin recurrence in the follow up period.

According to our results, the depth of tumor cells infiltration is also a significant factor in the prediction of contralateral metastasis (*p* = 0.0038). The median depth of tumor infiltration was 3 mm in group 1, 6 mm in group 2A/B and 8.5 mm in group 2C. Nonetheless, the diameter of the tumor is statistically insignificant (*p* = 0.764) in our evaluation. Our findings related to depth of tumor infiltration is in concordance to the current statement in German guideline with the possibility of groin metastasis depending on depth of tumor infiltration: ≤ 1 mm; 0%; 1.1–2 mm, 7.6%; 2.1–3 mm, 8.3%; 3.1–5 mm, 26.7% and ˃ 5 mm, 34.2%, respectively [4]. The depth of tumor has also been proposed to be taken into consideration for the decision on the extent of surgery and further management of VC [25, 38]. Future research should aim for bigger sample size and evaluate the correlation between the depth of tumor cells infiltration and the risk of contralateral groin metastasis. In addition, perineural invasion (PVI) has been reported to be an unfavorable prognostic factor for the outcome of patients indicating a more aggressive behavior of VC. Therefore, adjuvant treatment has been suggested in those women [39]. However, in our study, only 3 of 30 women with unilateral positive SLN had PVI in the primary tumor. One woman was from group 2A (negative bilateral IFL) and 2 women belonged to group 1A (negative unilateral IFL). The follow up of all these women was uneventful up to 60 months suggesting that PVI is not an unfavorable prognostic factor in our cohort.

In the case of lateralized lesion, the removal of contralateral LNs in case of unilateral positive SLNB should be discussed with the patients in regards to its benefits, risks and possible side effects. According to our results, it may perhaps be omitted but due to the low number of lateralized lesions in our study (20%), future prospective evaluation of lateralized lesions in VC is warranted. The few cases with lateralized lesions in our cohort of women with unilateral positive SLN (6/30 women) is the limiting factor to draw clear conclusions regarding the impact of contralateral IFL. In comparison, Woelber et al. and Nica et al. did not specify the location of the tumors in their study, as to whether they were midline or lateralized [7, 8, 12]. We suspect that it might be possible that the majority of their study subjects had lateralized tumors. This might explain why their radical bilateral IFL results had not shown any contralateral non-sentinel metastasis in contrast to our findings.

Perhaps there will be an alternative treatment option to avoid morbidity of IFL: According to a recently published study GROINSS V-II, radiotherapy could replace IFL if the tumor diameter is < 4 cm and SLNB metastasis is < 2 mm. However, in the case of sentinel node metastasis of > 2 mm, radiotherapy is not a safe alternative of IFL [33]. In addition, there is currently an ongoing nationwide study of VC in Sweden with inclusion criteria primary tumor ≥ 4 cm, primary multifocal tumors or local recurrences, being an exclusion criterion so far. The results will be expected at the end of 2021 and this could change the current clinical approach of SLNB in primary VC [40].

VC may also be diagnosed in pregnancy, in our center 5 women were diagnosed and treated in pregnancy within the last 15 years. We performed SLNB in collaboration with our department of nuclear medicine also in pregnant women after extensive counseling regarding the advantages and risks of the technique and written consent of the women. According to the current recommondations [41–43], this procedure should be done after the end of the 14^th^ week of pregnancy (first trimester) to be safe for the fetus. In pregnancy, a lower dose of radioactive Tc-99^m^ should be injected using a short-treatment protocol (SLNB can be done two hours following injection with lowest possible dose). The half-life of technetium 99m is six hours. Prompt nodal removal can reduce the chance of systemic exposure, even though fetal exposure is considered low when technetium is injected locally in the peritumoral region [41–43]. Moreover, diagnosis of VC in pregnancy is often delayed. A systematic review showed that the time interval from the first medical visit until first diagnosis of VC was more than eight weeks (62.5%). The first reason is low suspicion due to the rare occurrence of VC in younger-aged women (70%), second is noncompliance of patients (30%), and third is potential risk of vulvar biopsy resulting in feto-maternal complications during pregnancy [43]. In comparison to all gynecological cancers in pregnancy, VC is in fact considered to have the least possible complications in patients who undergo biopsy and/or operation [41–43].

Our data showed comparable morbidity of IFL with the reported data in the literature in respect of infection, lymph cysts, and lymphedema of the legs being 21.3–35.4%, 11–40% and 14–48.8%, respectively [44].

Although the overall survival (OS) of the patients in group 1, group 2 A/B and group 2C with contralateral positive LNs in IFL after negative SLNB is statistically not significant (*p* = 0.623, log rank test with Mantel Cox) (*p* = 0.517, Gehan-Breslow-Wilcoxon test, Fig. 2), there is a visible trend towards decreased survival in the women of group 2C with contralateral positive lymph nodes in IFL (Fig. 2). Interestingly and also unexpectedly, none of the women of group 1 who received only unilateral IFL due to unilateral positive sentinel lymph nodes developed groin recurrence in the observation time of 60 months. Neither in the contralateral groin nor unilaterally. No comparable survival rates exist in the literature since in the study of Woelber et al. [7] and Nica et al. [8] patients with negative SLNB were compared to women with metastatic groin LNs.

The limitations of this study are retrospective nature of data analysis, loss of some patients in follow-up examinations beyond 12 months following initial VC diagnosis in our clinic because change of address/phone number or switch to a new local gynecologist. Some patients were initially diagnosed in 2018 resulting in short follow up time. A further weakness is the small sample size of patients with lateralized vulvar tumor location.

## Conclusion

From our current findings, we confirm that radical bilateral IFL should be offered in treatment management of primary VC with anterior midline lesion and unilateral SLN metastasis. This is based on the findings that 4/18 (22.2%) women with unilateral positive SLN were further diagnosed with contralateral positive nodes using IFL. In our study, the tumors of these four women were located in the midline. However, the need for radical bilateral IFL in cases of lateralized tumor with positive ipsilateral SLNB should be further evaluated. Furthermore, the depth of tumor infiltrating cells correlated significantly and positively with the incidence rate of groin metastasis (*p* = 0.0038). According to our experience, in case of pregnancy, a punch biopsy is necessary in the management of suspicious vulvar lesion, along with facultative SLNB and surgical resection methods in case of proven malignancy with comparable good outcome to non-pregnant women. The management of VC and SLNB should be performed in a high volume center with an experienced team in marking SLN and performing the adequate surgical procedure. Well conducted counseling of the patients outlining advantages but also potential oncological risks of this technique especially concerning rate of groin recurrence is critical.

## Abbreviations

B: Biopsy
ESGO: European Society of Gynecological Oncology
FIGO: International Federation of Gynecology and Obstetrics
GOG 173: Gynecologic Oncology Group 173
GROINSS-V: GROningen INternational Study on Sentinel nodes in Vulvar cancer
HIV: Human immunodeficiency virus
HPV: Human papilloma virus
IFL: Inguino-femorale lymphadenectomy/complete radical groin dissection
G: Gynecology
LN: Lymph node
NCCN: National Comprehensive Cancer Network
O: Obstetrics
OS: Overall survival
RCOG: Royal College of Obstetricians and Gynecologists
RKI: Robert Koch Institute
SEER: Surveillance, epidemiology, and end results program
SCC: Squamous cell cancer
SLN: Sentinel lymph node
T, N, M, G, R: Tumor, node, metastasis, grading, resection status
UHD: University Hospital of Düsseldorf
USA: United States of America
VC: Vulvar cancer
WP: Weeks of pregnancy

## Availability of data and materials

All patients were diagnosed with primary vulvar squamous cell carcinoma at the department of O&G, UHD between 2002 and 2018. The data cannot be shared publicly in according to data protection act (Datenschutzgesetz) of German regulation.
